# Vital Pulp Therapy of Permanent Teeth with Reversible or Irreversible Pulpitis: An Overview of the Literature

**DOI:** 10.3390/jcm11144016

**Published:** 2022-07-11

**Authors:** Flavia Iaculli, Francisco Javier Rodríguez-Lozano, Benjamín Briseño-Marroquín, Thomas Gerhard Wolf, Gianrico Spagnuolo, Sandro Rengo

**Affiliations:** 1 Department of Neuroscience and Reproductive and Odontostomatological Sciences, University of Naples “Federico II”, 80131 Naples, Italy; flavia.iaculli@unina.it (F.I.); sanrengo@unina.it (S.R.); 2 Department of Dermatology, Stomatology, Radiology and Physical Medicine, Morales Meseguer Hospital, Faculty of Medicine, University of Murcia, 30100 Murcia, Spain; fcojavier@um.es; 3 Department of Restorative, Preventive and Pediatric Dentistry, School of Dental Medicine, University of Bern, 3010 Bern, Switzerland; benjamin.briseno@zmk.unibe.ch (B.B.-M.); thomas.wolf@zmk.unibe.ch (T.G.W.)

**Keywords:** hydraulic cements, irreversible pulpitis, pain, reversible pulpitis, vital pulp therapy

## Abstract

Vital pulp therapy (VPT) has been recently proposed as an alternative approach even in symptomatic mature permanent teeth with deep caries’ lesions, aiming to maintain the pulp vitality over time and/or to avoid non-surgical root canal therapy (NSRCT). However, to date, the diagnosis of reversible or irreversible pulpitis is only based on clinical pain quantity and quality, without precisely reflecting the pulp inflammation status. Therefore, the aim of the present study was to provide an overview based on the current scientific literature to demonstrate the clinical effectiveness of VPT on mature permanent teeth, validating the use of hydraulic calcium silicate-based cements and their role in pain management. VPT may be successfully applied not only in mature permanent teeth diagnosed with reversible pulpitis, but also in permanent dental elements with signs and symptoms of irreversible pulpitis. Hydraulic cements showed favorable outcomes in terms of decrease of pro-inflammatory mediators and of post-operative pain. Pain plays a central role in the chance to perform VPT in mature permanent teeth, since it may be considered as a pre-operative diagnostic criterion as well as a treatment success parameter. In addition, proper assessment of pulp inflammation and choice of appropriate materials are key factors in enhancing VPT success.

## 1. Introduction

Vital pulp therapy (VPT) consists of different treatment strategies to maintain the integrity and health of the teeth and preserve pulpal vitality in case of deep caries lesions approximating/involving the pulp or in case of pulp tissue exposure due to trauma or mechanical causes [1]. VPT basically includes direct and indirect pulp capping and pulpotomy procedures such as partial and complete pulpotomy; it is successfully performed in daily treatment of primary teeth (to increase their maintenance over time) and of immature permanent elements to allow apexogenesis [2]. Recent findings proposed VPT for broader focus and as an alternative approach even in symptomatic mature permanent teeth with deep caries lesions, aiming to maintain the pulp vitality over time and/or to avoid non-surgical root canal therapy (NSRCT) [3,4]. However, the outcomes and success of VPT are strictly related to the pulp inflammation severity and the histopathological involvement of pulp tissue [5]. Furthermore, it has been demonstrated that there is no precise correlation between clinical symptoms and the histopathological status of the pulp, mainly in case of irreversible pulpitis, that might lead to a wrong diagnosis [6,7]. Unfortunately, to date, the reversible or irreversible pulpitis diagnosis is based on anamnesis, subjective clinical pain parameters, and pulp sensibility testing without reflecting the actual pulp inflammation status [5].

Therefore, the present study aimed to provide an overview based on the current scientific literature to:

- improve the diagnosis of pulp health status;
- demonstrate the clinical effectiveness of VPT on mature permanent teeth;
- support the use of hydraulic cements during VPT;
- evaluate pain as a potential diagnostic criterion.

## 2. Diagnosis of Reversible/Irreversible Pulpitis

**Figure 1:**
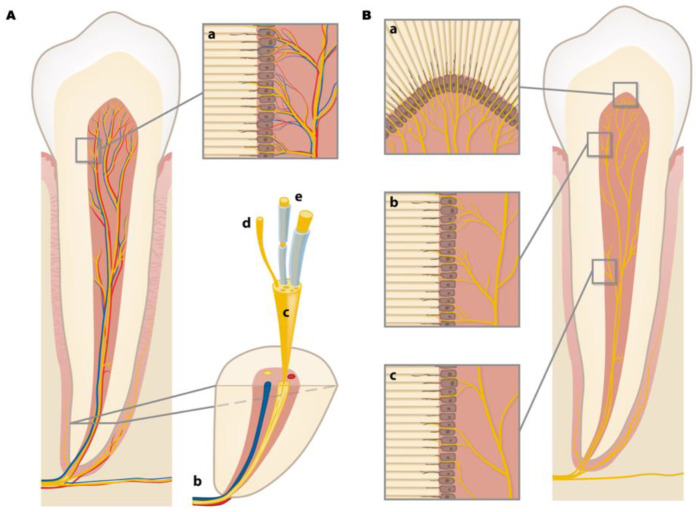
(**A**) Pulp-dentinal complex vascularization and innervation. (a) blood vessels (red and blue) and sensory nerves (yellow) from pulp chamber to dentinal tubules forming the plexus of Raschkow; (b) neurovascular bundle at tooth apex; (c) nerve bundle; (d) unmyelinated nerve fiber; (e) myelinated nerve fibers; (**B**) tooth innervation; (a) the coronal aspect of the pulp chamber presents highly packed nerve terminals that progressively decrease in cervical (b) and apical direction (c) nerve terminals both end in the odontoblastic layer and within the dentinal tubules.

Two distinct nerve pathways are responsible for dental pulp pain [8]. Myelinated A-delta fibers (A∂ fibers) are involved in fast pain conduction, causing a rapid, sharp, and localized pain response. In case of a cold stimulus application to tooth crown, a rapid A∂ pain response is evoked, followed by an almost immediate pain cessation [8]. Unmyelinated C-fibers are responsible for a slow pain conduction, difficult to localize, and mostly caused by a heat stimulus (Figure 1A,B). The pain response is rapid and brief; ceasing after a short time, more intense pain with a greater frequency is evoked [8]. Dental pain caused by A-delta fibers is more likely to be addressed by diagnostic vitality tests, whereas pain caused by C-fibers is usually associated with pulp tissue pathological inflammation. In the absence of external triggers, pain related to inflamed or injured tissues may occur spontaneously [9,10].

**Figure 2:**
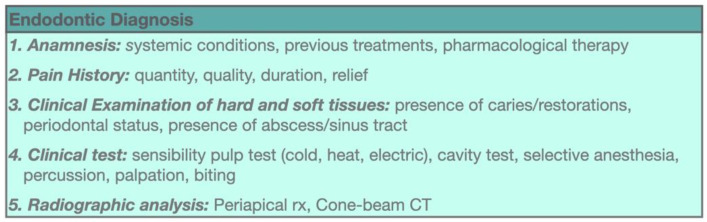
Endodontic diagnosis procedure.

**Figure 3:**
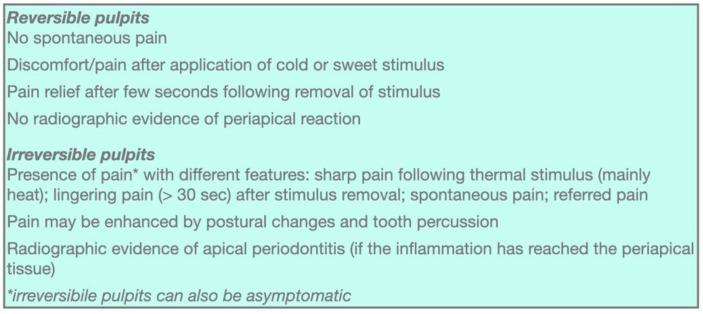
Signs and symptoms of pulpitis.

At present, the severity of pulp inflammation can be clinically assessed only by soft and hard tissue examination, with subjective interindividual patient’s pulp sensitivity test responses and radiological examination (Figure 2). The pulpal inflammation degree is often determined after applying a cold stimulus that can induce an exaggerated and “lingering” response due to C-fiber sensitization and inflammation-induced hypersensitivity [3]. However, when considering these diagnostic criteria, a quantitative pain evaluation to be able to reliably differentiate between pain induced by reversible or irreversible pulp inflammation is not possible [11]. From a biological point of view, the difference between reversible and irreversible pulpitis is related to specific in situ inflammatory mediator expression levels [5]. Even though pain is one of the main symptoms of infection/inflammation, its presence or intensity does not precisely correlate with the pulp tissue involvement extent and histopathological status severity [7]. Indeed, it should be stressed that a pulpitis may often be asymptomatic [12] (Figure 3).

Caries-related irreversible pulpitis is often histologically characterized by necrotic tissue colonized by bacteria and sometimes micro-abscesses formation and healing inability. On the other hand, the surrounding pulp tissue develops an immune defense reaction to prevent infection from spreading and is to a great extent free of inflammation or necrosis [7,13]. Accordingly, if isolation and removal of the infected pulp (prior to necrosis spread) would be possible, the healthy non-infected pulp tissue portion would remain healthy [6]. Conversely, when the pulp is only reversibly inflamed, it has a healing and tissue repair potential. Even though reversible and irreversible pulpitis can be histologically differentiated [14], pulp tissue repair cannot be objectively clinically appreciated. Indeed, Ricucci et al. [14] reported a higher correlation between clinical diagnosis of normal pulp/reversible pulpitis and the histologic findings than irreversible pulpitis cases, representing an additional issue in the therapeutic choice. Therefore, a biological-based pulp examination is needed to allow a precise pulp status diagnosis, thus accurately determining a related clinical approach that will increase the treatment prognosis of a pulp inflamed tooth.

## 3. Vital Pulp Therapy in Mature Permanent Teeth

Considering the potential, even partially, of the inflamed pulp to heal, VPT could be regarded as a reliable alternative to NSRCT in mature teeth with carious lesions affecting the pulp, regardless of a reversible/irreversible pulpitis diagnose [3,4]. In this regard, several authors have compared the outcomes of pulpotomy and traditional endodontic treatment on permanent teeth over time. Two systematic reviews on the topic were recently published [15,16]. Briefly, Cushley et al. [15] demonstrated that a complete coronal pulpotomy was effective in treating permanent teeth affected by carious-related irreversible pulpitis showing clinical and radiographic success rates of 97.4% and 95.4%, respectively, after 12 months of follow-up. At 36-month recall visits, the clinical and radiographic success rates decreased to 93.97% and 88.39%, respectively, suggesting a comparable success with NSRCT over time. Thus, the authors concluded that coronal pulpotomy of symptomatic permanent teeth could be a potential alternative to NSRCT and should not only be considered as an emergency pain relief procedure before conventional endodontic therapy [15]. Santos et al. [16] reported a success range between 81 and 90% obtained by VPT performed with hydraulic calcium silicate cements in permanent mature posterior teeth with symptomatic irreversible pulpitis, even though two of the included randomized clinical trials reported comparable results between VPT and NSRCT [16]. Particularly, a 5-year randomized clinical trial included in both systematic reviews assessing VPT using a calcium-enriched mixture cement (CEM) and NSRCT of 271 mature molars clinically diagnosed with irreversible pulpitis revealed no significant difference in the treatment outcomes with success rates of 78.1% and 75.3% for the VPT/CEM and NSRCT groups, respectively [17]. Another included randomized controlled trial [18] compared MTA pulpotomies and NSRCTs of symptomatic permanent teeth with deep caries lesions after 18-months and reported no significant differences between the groups with an overall success of 85% in the MTA pulpotomy and 87.5% in the NSRCT groups. Moreover, the same study reported that the pulpotomy group revealed a statistically significant lower pain within the first-week post-intervention than the NSRCT group. Recently, Koli et al. [19] suggested a combination of nonsurgical endodontic therapy and VPT as a treatment option for mature permanent mandibular molars with symptomatic irreversible pulpitis and apical periodontitis, demonstrating a slightly higher success rate than conventional NSRCT. However, it should be stressed that both reviews [15,16] included studies with limitations and potential bias; particularly, Cushley et al. [15] included not only randomized clinical trials and prospective studies, but also retrospective papers dealing with just one treatment approach as “full pulpotomy”, excluding some papers that might potentially fulfill the established inclusion criteria. On the other hand, Santos et al. [16] reported a higher level of evidence, accepting only prospective clinical studies and randomized clinical trials that included both partial and full pulpotomy. This aspect might provide different results in terms of success and survival rates; indeed, better outcomes were observed for partial pulpotomy when compared to a full procedure [16]. However, further randomized controlled trials with a larger sample size and longer follow-up period should be prospectively conducted to support the effectiveness of the VPT approach as an alternative to NSRCT. The lack of univocal clinical indications, technical procedures, outcome assessment and adequate sample size of the studies provide limited evidence that should be additionally acknowledged in future studies. The clinical effectiveness of VPT on mature permanent teeth should be deeply established to represent a reliable therapeutic option to clinicians.

## 4. Hydraulic Materials in VPT and Effect on Pain Management

Hydraulic tri-/dicalcium silicate cements have been shown to yield a reliable long-term VPT outcome when compared with calcium hydroxide in cariously exposed pulps of permanent teeth [20,21,22]. Indeed, current tri-/dicalcium silicate materials seem to provide a clinically sufficient sealing of exposed pulps, avoiding bacterial micro-leakage, thus supporting VPTs as a definitive treatment modality even in mature permanent teeth affected by pulpitis [15,23].

Calcium silicate-based materials own advantageous physical and biological features such as interactivity (Ca and OH ions release and production of higher alkalinity), apatite-forming stimulation, biocompatibility and a bactericidal effect [3,24]. The release of Ca and OH ions promotes cell differentiation and proliferation, wound healing, tissue repair, and hard tissue mineralization (dentinal bridge formation), thus mantling the pulp tissue vitality [20,25]. It has been reported [26] that calcium silicate-based materials have the potential to decrease the pro-inflammatory mediator expression and the occurrence of post-operative pain [3,27]. From a clinical point of view, the employment of Biodentine after a VPT in permanent teeth with irreversible pulpitis and apical periodontitis showed 100% clinical and 98.4% radiographic success after one year [28] and 100% after two years of follow-up [29]. Comparable results were also obtained with MTA in VPT procedures of permanent molars affected by irreversible pulpitis [30]. Accordingly, a systematic review evaluating VPTs in permanent mature teeth with symptomatic irreversible pulpitis [16] reported an overall success rate (3–5 years) of 85% and 90%, with MTA and Biodentine, respectively. The same authors [16] reported non-promising clinical and radiographical results of VPTs when employing calcium hydroxide. These results (55% of success using calcium hydroxide vs. >85% using calcium-silicate cements) support a clinically safe and successful VPT procedures on mature symptomatic teeth when using hydraulic tri-/dicalcium silicate cements, although the possibility of a high bias risk of some of the studies included in the aforementioned systematic review [16] should always be kept in mind. Comparing the effectiveness of three calcium silicate-based materials (ProRoot MTA, Biodentine, and TotalFill) used in full pulpotomies of mature permanent molars [31], the pain relief was about 97% within the first week post-operatively regardless of the materials. The 12-month success revealed an overall success of 92.3% with no significant difference among groups (91.8% in MTA vs. 93.3% in Biodentine vs. 91.9% in TotalFill). These results corroborate the promising histologic outcomes of the same hydraulic calcium silicate-based cements when used as capping materials in full pulpotomy [32].

A complete pain relief or mild pain was reported in 97% of the subjects (n = 90) underwent full pulpotomy using hydraulic cement on symptomatic mature permanent teeth (n = 100), during the first week after treatment [33]. In addition, the same study showed an 83.8% of success after 4 years of follow-up; however, out of 23/100 cases that had failed, only 10 were classified as endodontic failure, so the clinical success might be assumed to be close to 90% [33].

Concerning post-operative pain outcomes, it remains controversial whether the low pain level after VPT with calcium silicate-based materials is due to the chemical and/or biological properties of the materials or is due to the absence of RCT procedures, that in turn allow the extrusion of debris, irrigants, cement into the periradicular tissues and over-instrumentation [34,35]. A recent study comparing postoperative pain after NSRCT or full pulpotomy with MTA or CEM in mature permanent teeth [36] reported a considerable decrease after 24 h among all groups (NSRCT: from 56.5% to 13.1%; MTA: from 55.7%, to 10.6%; CEM: from 56.7% to 12.9%) demonstrating comparable postoperative pain relief. However, the study failed to provide the superiority of one procedure to another in terms of pain management. On our concern, pain prevention should be carefully considered as a research parameter in future studies to be able to understand if there is an additional advantage to perform VPT in mature permanent teeth with symptomatic pulpitis when compared with NSRCT.

## 5. Discussion

VPT is basically focused on the concept that there is no best root canal filling material than vital pulp [37]. Dental pulp tissue has a reparative potential that is crucial during the healing process of an amputated pulp, even in the case of irreversible pulpitis [7,38]. Once the inflamed/infected pulp is removed, the repair potential of the healthy radicular pulp is preserved; thus, the tooth vitality is, at least to a certain extent, maintained [7]. Moreover, if the amputated pulp tissue is sealed by a biocompatible material that prevents (bacterial) micro-leakage [39,40], the clinical success of the treatment over time should be comparable with the one obtained with a conventional endodontic treatment. However, to date, due to a limited VPT treatment outcome, data regarding mature permanent symptomatic teeth and, partially, due to the clinical diagnosis inaccuracy, there is the tendency to completely remove the pulp tissue to control postoperative re-infection and thus the pain as well [5]. The diagnosis of pulpal diseases is based on several parameters such as a patient’s subjective individual pain perception, objective/subjective clinical examination and radiographic findings, without considering the histopathological status of the pulp tissue and, mainly, its healing reparative potential [4,14].

Under this perspective, the diagnosis of irreversible pulpitis should be reviewed, considering not only the clinical signs and symptoms but also the pulp tissue inflammation involvement; furthermore, the concept that an “inflamed pulp is incapable of healing” should be thoroughly revised [3,4]. Practitioners need new diagnostic tools to support a clinical treatment decision that would allow them to consider VPT as a reliable alternative [41]. To date, the clinicians have the unique choice to perform pulpotomy procedures if bleeding is controlled within a 2–5 min interval of time and there are no signs of acute inflammation [6]. However, Santos et al. [16] reported, in a recent systematic review, that the time to control bleeding ranged from 2 to 20 min and concluded that its influence on the outcome of VPT is still ambiguous and under debate.

Pain plays a decisive role in this issue since it is currently considered as a pre-operative diagnostic as well as post-operative success/unsuccess diagnostic criterion [33,42,43]. However, the relationship of preoperative pain and its intensity as well as the degree of pulp inflammation to the occurrence of postoperative pain or to disease progression are still not reliable predicting factors [44]. The use of hydraulic calcium silicate-based cements in VPTs has shown promising clinical results in decreasing the pulp tissue inflammation level and increasing the pulp healing; however, their role in the reduction of post-operative pain [45,46] or their benefit on pain management remains controversial and should be investigated in detail.

The ability to accurately and objectively diagnose the true inflammatory state of the pulp and the lack of consensus in decision-making represent two major issues in the chance to perform VPT on permanent teeth [47]. In addition, VPT presents a low cost/effectiveness ratio and requires a specific tissue handling and management that could represent a clinical limit [48].

Biological-based pulp tissue diagnosis is of crucial importance to be able to understand and categorize reversible/irreversible pulpal diseases and to select a treatment decision on whether to perform a VPT instead of NSRCT with a high level of success confidence [5]. Further scientific evidence should be provided to support the biological and economic advantages of VPTs over traditional endodontic treatment of permanent mature teeth with irreversible pulpitis.

## 6. Conclusions

Within the limitation of the present paper, it can be concluded that:

- VPT may be successfully applied in mature permanent teeth diagnosed with reversible pulpitis or even with signs and symptoms of irreversible pulpitis;
- Adequate pulp inflammation diagnoses and selection of appropriate materials, such as bioactive cements, are key factors in enhancing VPT success;
- Pain may be considered as a pre-operative diagnostic criterion as well as a treatment success parameter. However, further studies are needed to evaluate its role in the progression of pulp disease and the potential benefit obtained using hydraulic materials.

## Data Availability

Not applicable.
